# Supplementary material for: The SUN-family protein Sad1 mediates heterochromatin spatial organization through interaction with histone H2A-H2B
Source: Nat Commun. 2024 May 21;15:4322. doi: 10.1038/s41467-024-48418-7 (PMC11109203; doi:10.1038/s41467-024-48418-7)
Supplement: Supplementary file 1 — Supplementary Information [file 41467_2024_48418_MOESM1_ESM.pdf]

Supplementary information

The SUN-family protein Sad1 mediates heterochromatin spatial organization through interaction with histone H2A-H2B

Wenqi Sun[1,2,6], Qianhua Dong[3,6], Xueqing Li[1,2,6], Jinxin Gao[3,6], Xianwen Ye[2,4], Chunyi Hu[5], Fei Li[3,*], Yong Chen[1,2,4,*]

[1]State Key Laboratory of Molecular Biology, Key Laboratory of Epigenetic Regulation and Intervention, Shanghai Institute of Biochemistry and Cell Biology, Center for Excellence in Molecular Cell Science, Chinese Academy of Sciences, Shanghai, 200031, China.

[2]University of Chinese Academy of Sciences, Beijing 100049, China.

[3]Department of Biology, New York University, New York, NY 10003, USA.

[4]School of Life Science and Technology, ShanghaiTech University, 100 Haike Road, Shanghai 201210, China.

[5]Department of Biological Sciences, Faculty of Science, National University of Singapore, Singapore 117543.

[6]These authors contributed equally to this work.

*Correspondence: yongchen@sibcb.ac.cn and fl43@nyu.edu

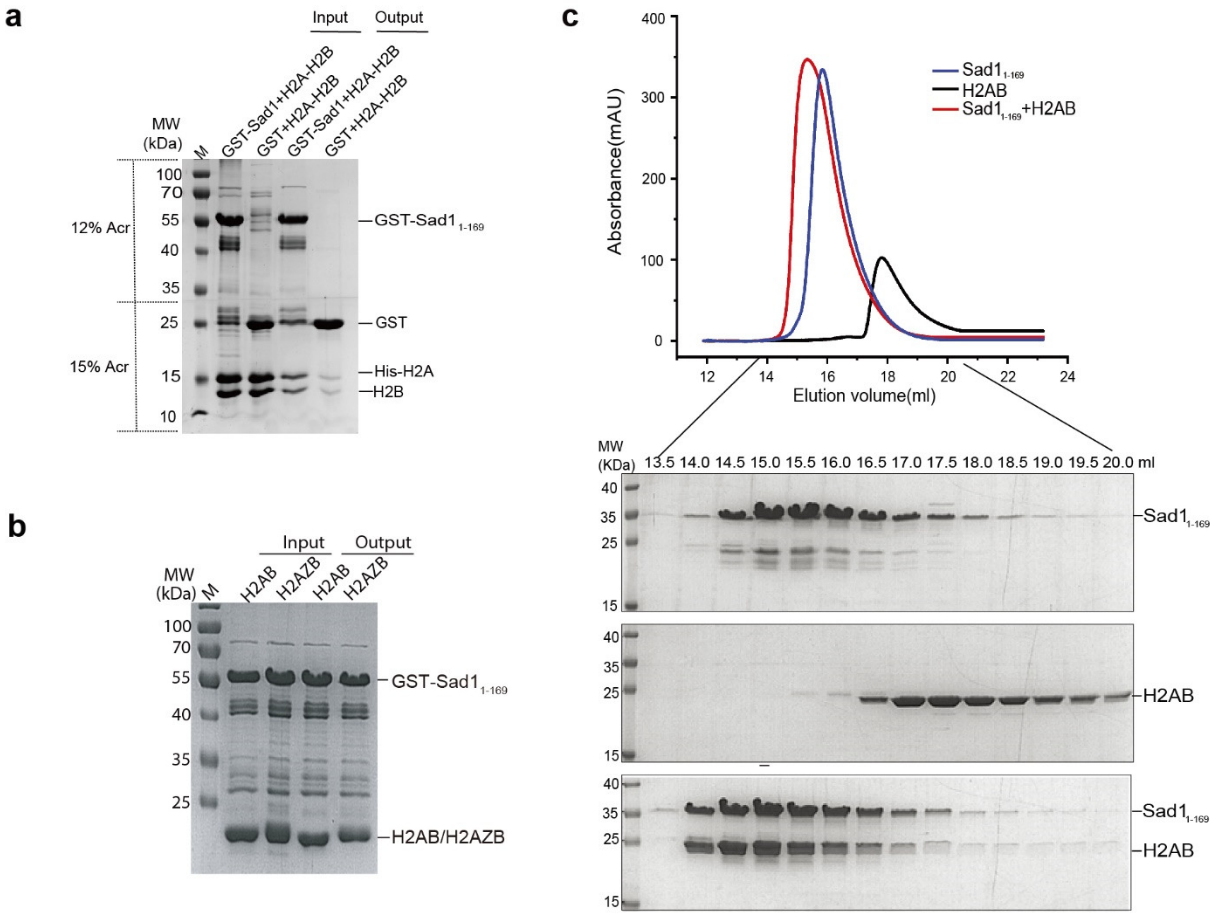

Supplementary Figure 1. Identify the interaction between Sad1 and H2A-H2B.

a. GST pull-down assays showed that although His-H2A-H2B has some non-specific binding to beads, GST-Sad1$_{N169}$ could pull down much more His-H2A-H2B than GST. A home-made segmented gradient gel was used. The acrylamide concentration is 12% for the top half and 15% for the bottom half. Source data are provided as a Source Data file.

b. GST pull-down assays showed that GST-Sad1$_{1-169}$ could pull down similar amounts of H2AB and H2AZB. H2AB is a single-chain fusion of H2A and H2B. H2AZB is a single-chain fusion of H2A.Z and H2B. A regular gel with 12% acrylamide concentration was used. Source data are provided as a Source Data file.

c. The interaction between Sad1 and H2AB was confirmed by size-exclusion chromatography. Top: the gel-filtration profiles for Sad1 only, H2AB only, and the Sad1-H2AB complex. Bottom: Coomassie brilliant blue staining of gel-filtration fractions.

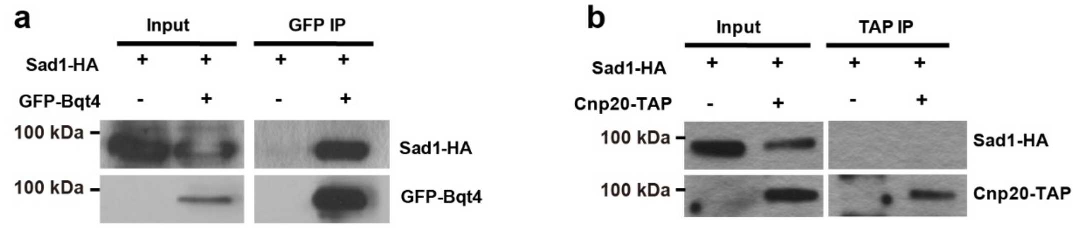

Supplementary Figure 2. Positive and negative Co-IP controls to verify the interactions between Sad1 and histone H2B.

a. Co-immunoprecipitation assays showed that Sad1 and Bqt4 co-existed in a complex. Lysates from the indicated cells were immunoprecipitated with an antibody specific to GFP. Immunoprecipitated samples were analyzed by immunoblotting using the indicated antibodies. Source data are provided as a Source Data file.

b. Co-immunoprecipitation assays showed that Sad1 and Cnp20 did not co-exist in a complex. Lysates from the indicated cells were immunoprecipitated with an antibody specific to TAP. Immunoprecipitated samples were analyzed by immunoblotting using the indicated antibodies. Source data are provided as a Source Data file.

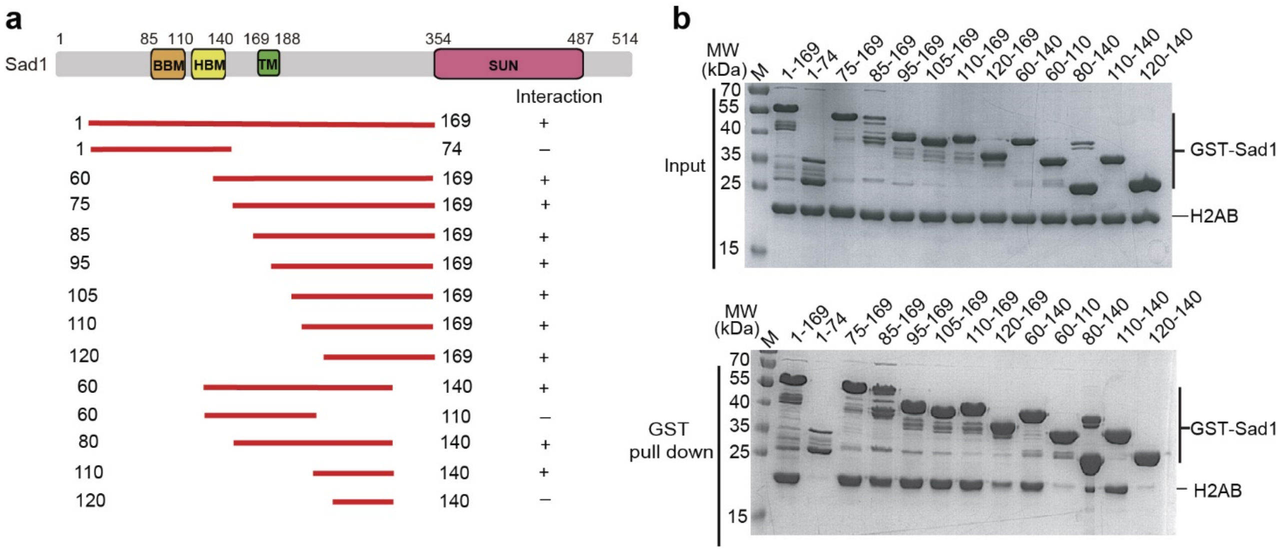

Supplementary Figure 3. Mapping the minimal interaction region between Sad1 and H2AB.

a. Domains organization of Sad1: TM, transmembrane helix; SUN, Sad1-UNC84 homology domain; BBM, Bqt4 binding motif; HBM, histone binding motif.

b. GST pull-down assays showed that the minimum histone binding motif (HBM) on Sad1 was from 110 to 140. A regular gel with 12% acrylamide concentration was used. Source data are provided as a Source Data file.

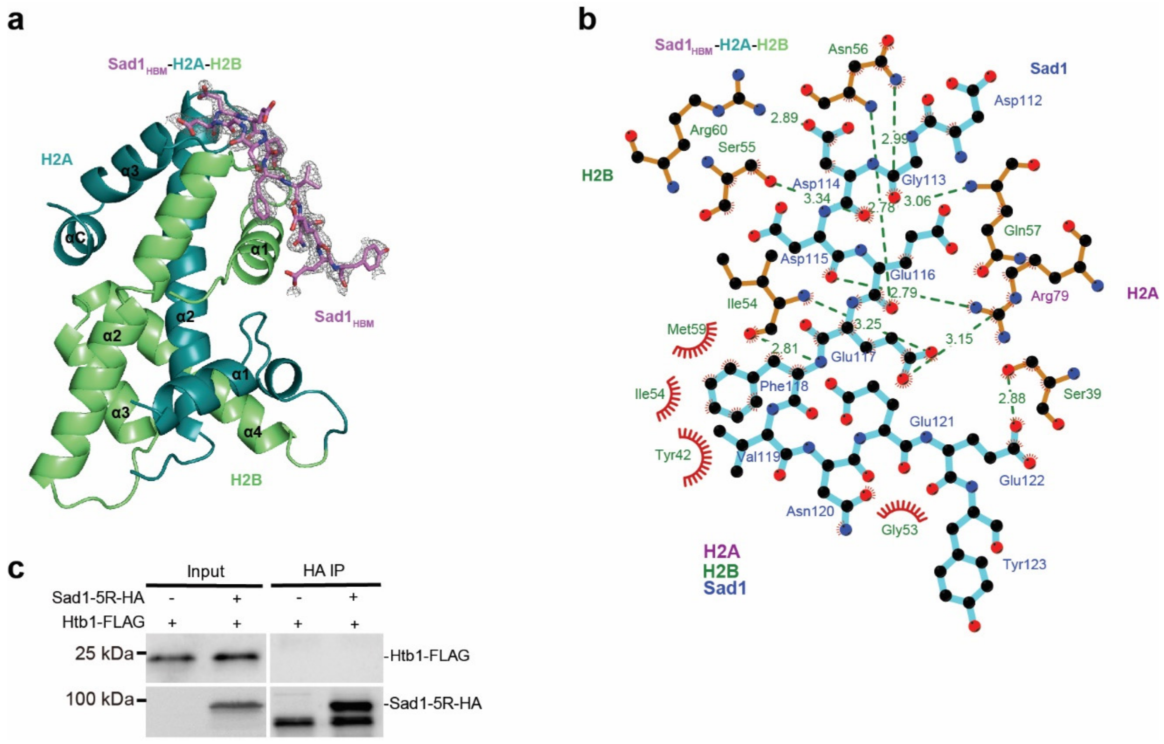

Supplementary Figure 4. Structural analysis of H2AB in complex with Sad1$_{HBM}$ and H2AB.

a. The overall structure of the Sad1$_{HBM}$-H2AB complex. The omit map is shown for Sad1$_{HBM}$ at contour level 3σ.

b. The details of both electrostatic and hydrophobic interactions between Sad1$_{HBM}$ and H2AB. Hydrogen bonds are shown as dashed green lines.

c. Co-immunoprecipitation assays showed that the Sad1-5R mutant is unable to interact with histone H2B^{Htb1}. Lysates from cells carrying Sad1-5R-HA and H2B^{Htb1}-FLAG were immunoprecipitated with an antibody specific for HA. Immunoprecipitated samples were analyzed by immunoblotting using anti-FLAG and anti-HA antibodies. Cells expressing H2B^{Htb1}-FLAG were used as a control. Source data are provided as a Source Data file.

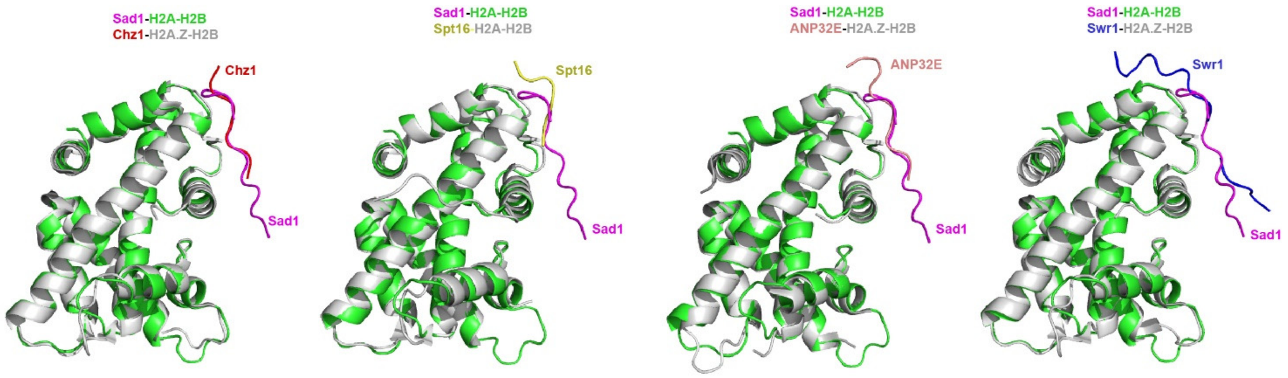

Supplementary Figure 5. Structural comparison of the Sad1$_{HBM}$-H2AB complex with other H2A-H2B complexes. Chz1-H2A.Z-H2B (PDB: 6AE8), Spt16-H2A-H2B (PDB: 4WNN), ANP32E-H2A.Z-H2B (PDB: 4CAY), and Swr1-H2A.Z-H2B (PDB: 4M6B). The histone-binding peptides in different complexes show similar configurations.

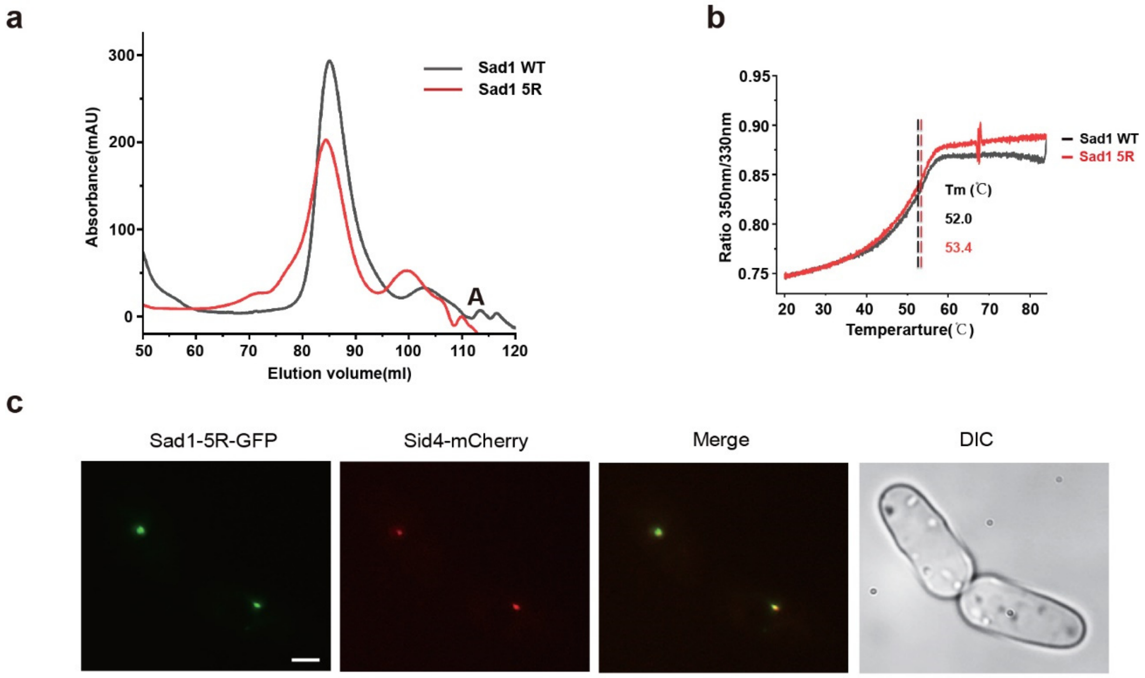

Supplementary Figure 6. Sad1-5R mutation does not affect protein folding and its SPB localization.

a. The Sad1$_{1-169}$-5R mutant protein has a similar gel-filtration profile on Superdex Hiload 200 as that of WT Sad1$_{1-169}$, indicating that the 5R mutation does not affect the overall structure of Sad1.

b. NanoDSF analyses show that the Sad1$_{1-169}$-5R mutant protein has a similar thermal melting curve as that of WT Sad1$_{1-169}$. The Y axis is the ratio of intrinsic fluorescence emission 350 nm/330 nm. The dashed lines indicate the melting temperature (Tm).

c. Representative images showed that Sad1-5R-GFP colocalizes with the SPB protein, Sid4-mCherry. Scale bars, 2 μm.

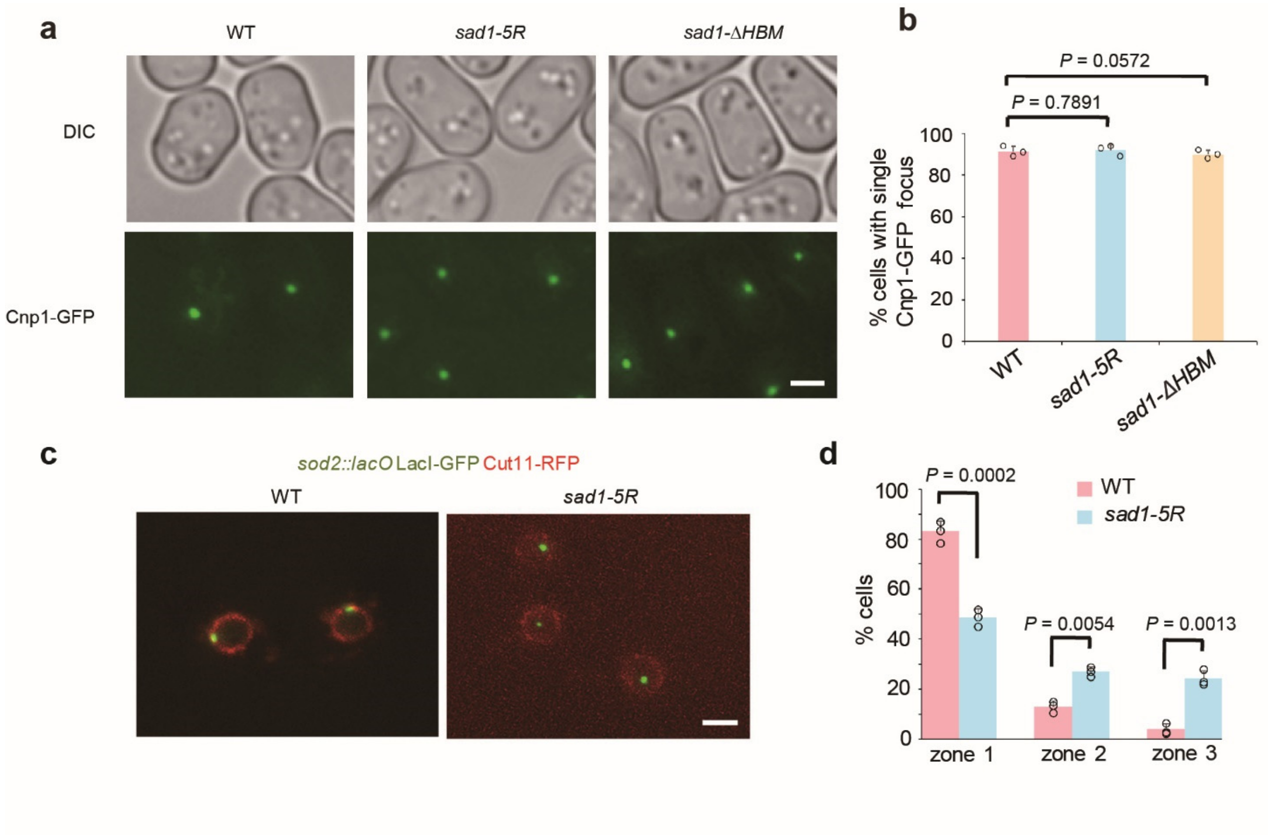

Supplementary Figure 7. Sad1 mutants do not affect centromere clustering at SPB, but affect telomere-NE association.

a. The localization of centromeres (represented by Cnp1-GFP) in the interphase was not disrupted by Sad1 mutants, indicating that Sad1-histone interaction is not important for centromere localization to SPB.

b. Quantification of the percentage of cells showing a single focus of Cnp1-GFP. Three independent experiments were repeated. 100 cells were scored in one single experiment. Data are presented as mean ± SD. The indicated P values are from two-sided Student's *t* test. Source data are provided as a Source Data file.

c. Representative images showed the telomere localization using a telomere mark (*sod2::lacO/*LacI-GFP) in the indicated cells. Scale bar, 2 µm.

d. Percentage of cells with telomeres positioned in three concentric zones. Three independent experiments were repeated. 200 cells carrying *sod2::lacO/*LacI-GFP and Cut11-RFP were scored in one single experiment. Data are presented as mean ± SD. The indicated P values are from two-sided Student's *t* test. Source data are provided as a Source Data file.

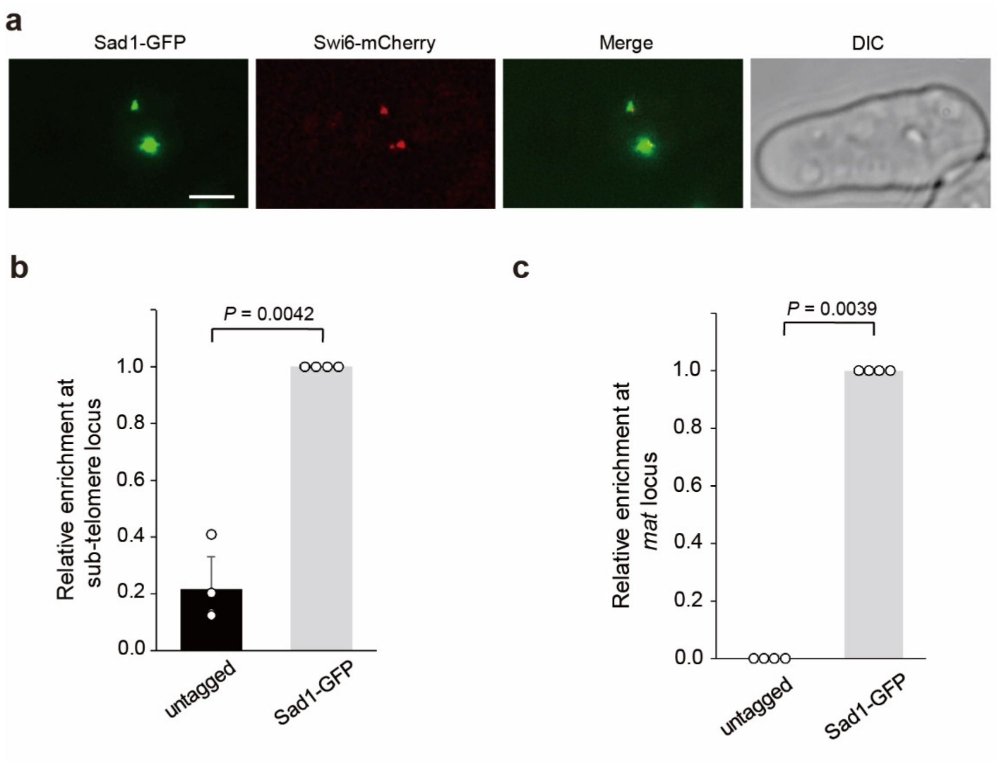

Supplementary Figure 8. Sad1 associates with heterochromatin regions.

a. Representative images showed that Sad1-GFP on the nuclear envelope colocalizes with the heterochromatin protein, Swi6-mCherry. Scale bars, 2 µm.

b. ChIP-qPCR analysis of Sad1-GFP in sub-telomere region. ChIP assays were performed from extracts of cells expressing Sad1-GFP using an anti-GFP antibody. Untagged cells were used a negative control. Immunoprecipitated DNAs were quantified by RT-qPCR using primers specific for the sub-telomere and a control gene, *act1⁺*. The fold of enrichment was normalized to *act1⁺* and calculated by comparative ΔCt of IP with whole cell extracts. The level of Sad1-GFP was set to 1. Data are presented as mean ± SD from n=4 independent experiments. The indicated P value is from two-sided Student's *t* test. Source data are provided as a Source Data file.

c. ChIP-qPCR analysis of Sad1-GFP in the *mat* locus. ChIP assays were performed from extracts of cells expressing Sad1-GFP using an anti-GFP antibody. Untagged cells were used a negative control. Immunoprecipitated DNAs were quantified by RT-qPCR using primers specific for the *mat* locus and a control gene, *act1⁺*. The fold of enrichment was normalized to *act1⁺* and calculated by comparative ΔCt of IP with whole cell extracts. The level of Sad1-GFP was set to 1. Data are presented as mean ± SD from n=4 independent experiments. The indicated P value is from two-sided Student's *t* test. Source data are provided as a Source Data file.

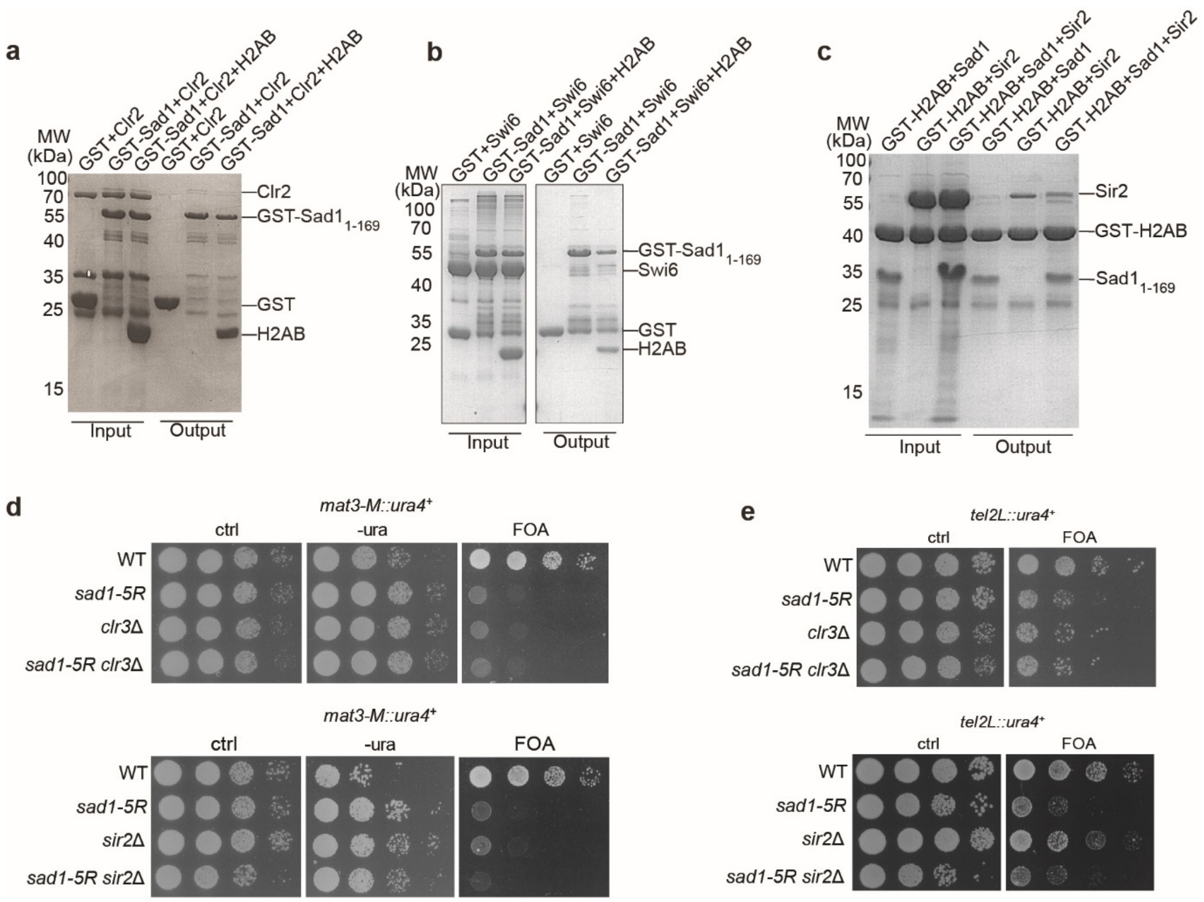

Supplementary Figure 9. The interactions between Sad1 and HDACs.

a. GST pull-down assays showed that Sad1 could not interact with Clr2 in the absence or presence of H2AB. Source data are provided as a Source Data file.

b. GST pull-down assays showed that Sad1 could not interact with Swi6 in the absence or presence of H2AB. Source data are provided as a Source Data file.

c. GST pull-down assays showed that both Sir2 and Sad1 could bind GST-H2AB independently. H2AB may function as a bridge to interact with Sir2 and Sad1 simultaneously to form a complex. Source data are provided as a Source Data file.

d. Silencing of the mating-type locus in the indicated strains was analyzed using an *ura4*$^+$ reporter inserted in the mating-type locus in indicated media. -ura, no uracil. Ctrl. Control.

e. Silencing of the telomere locus in the indicated strains was analyzed using an *ura4*$^+$ reporter inserted in the telomere locus in indicated media. -ura, no uracil. Ctrl. Control.

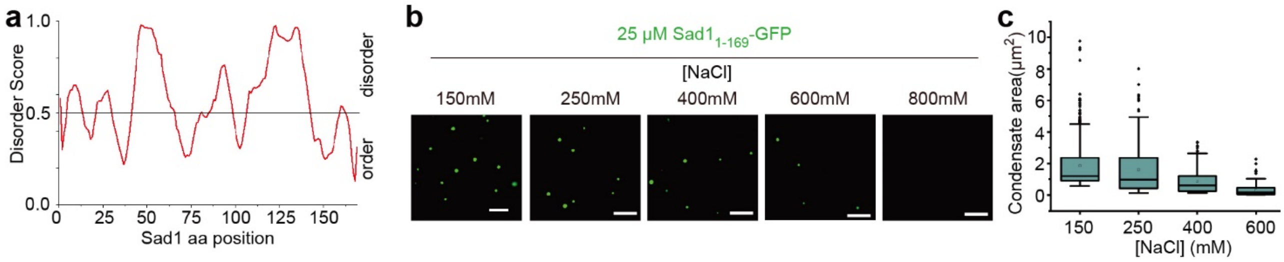

Supplementary Figure 10. Phase separation property of Sad1$_{1\text{-}169}$.

a. Graph of intrinsic disorder of Sad1$_{1\text{-}169}$ as calculated by PONDR (Predictor of Natural Disordered Regions) (http://pondr.com/).

b. Representative images of droplet formation of Sad1$_{1\text{-}169}$-GFP (25 µM) at different NaCl concentrations. The scale bar is 10 µm.

c. Quantitation of condensate area for Sad1$_{1\text{-}169}$-GFP (25 µM) at different NaCl concentrations. The droplets can be disrupted by higher salt concentrations (>600 mM NaCl). The numbers of droplets used for quantitation: 150 mM NaCl, n=488; 250 mM NaCl, n=143; 400 mM NaCl, n=152; 600 mM NaCl, n=133. Data are presented as box plots. Minima: Lower limit of the whisker; Maxima: Upper limit of the whisker; Centre: Median line inside the box; The upper and lower box bounds represent the 25% and 75% percentile of data. Black squares are outliers. Source data are provided as a Source Data file.

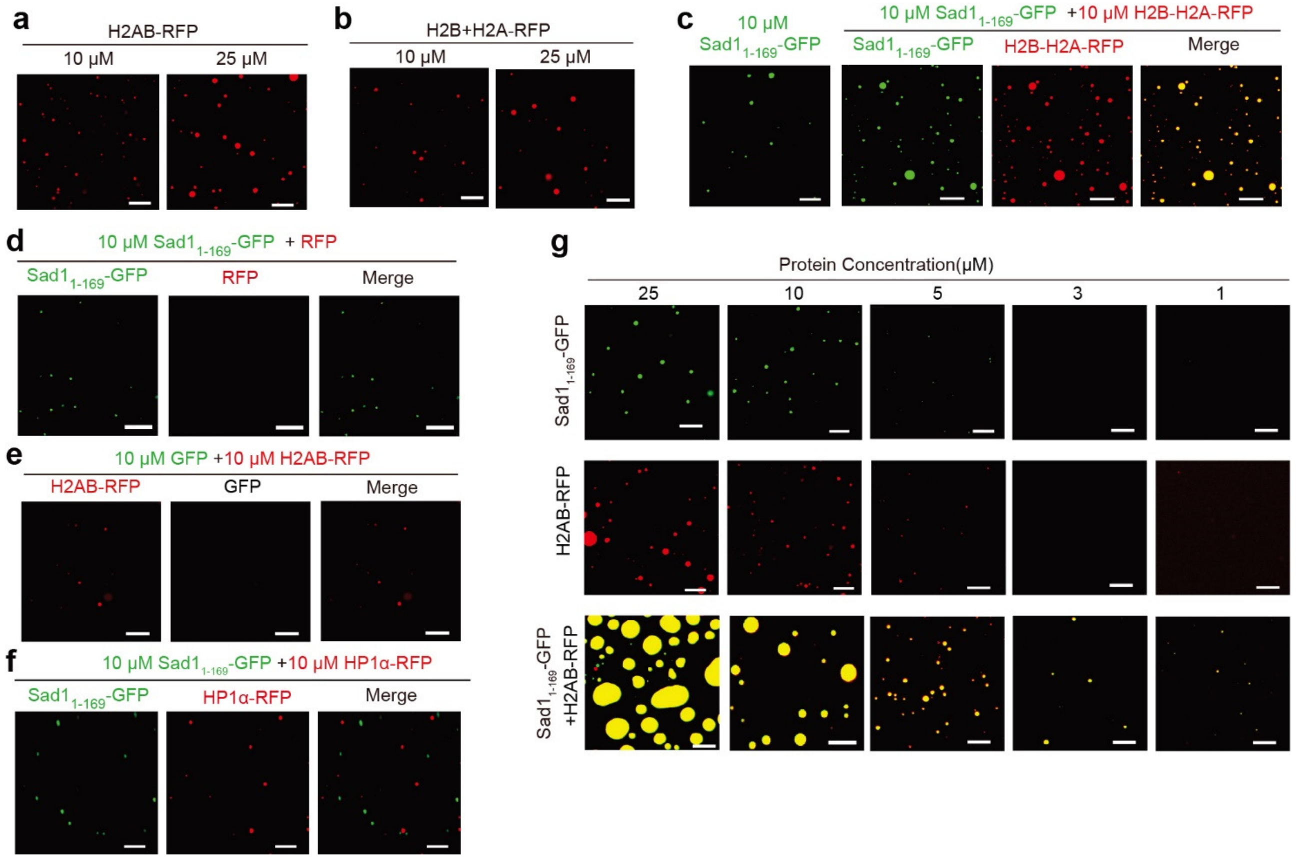

Supplementary Figure 11. H2A-H2B enhances the phase separation of Sad1$_{1\text{-}169}$.

a. Representative images of droplet formation of the H2AB-RFP fusion proteins at different concentrations in droplet formation buffer. H2AB stands for the fusion protein of H2B and H2A. The scale bar indicates 10 μm and applies to all the subsequent images

b. Representative images of droplet formation of the heterodimer composed of the full-length H2B and H2A-RFP. The H2A-H2B heterodimer also undergoes similar LLPS as the H2AB fusion protein.

c. Representative images of Sad1$_{1\text{-}169}$-GFP (10 μM) mixed with H2B+H2A-RFP (10 μM). The presence of H2A-H2B heterodimer also increased the droplet size of Sad1$_{1\text{-}169}$.

d. Fluorescence images of the mixture of Sad1$_{1\text{-}169}$-GFP (10 μM) and RFP (10 μM) in droplet formation buffer. RFP itself cannot be incorporated into Sad1$_{1\text{-}169}$-GFP droplets and cannot increase the size of Sad1$_{1\text{-}169}$-GFP droplets.

e. Fluorescence images of the mixture of H2AB-RFP (10 μM) and GFP (10 μM) in droplet formation buffer. GFP itself cannot be incorporated into H2AB-RFP droplets and cannot increase the size of H2AB-RFP droplets.

f. Fluorescence images of the mixture of Sad1$_{1\text{-}169}$-GFP (10 μM) and HP1-RFP (10 μM) in droplet

formation buffer. Although both Sad1$_{1-169}$-GFP (10 μM) and HP1-RFP (10 μM) can form droplets, these two droplets do not fuse and do not affect the droplet size of each other.

g. H2AB lowers the critical concentration required for the phase separation of Sad1$_{1-169}$-GFP. Sad1$_{1-169}$-GFP alone forms droplets at a concentration above 5μM, while Sad1$_{1-169}$-GFP and H2AB-RFP complex can form droplets as low as 1μM.

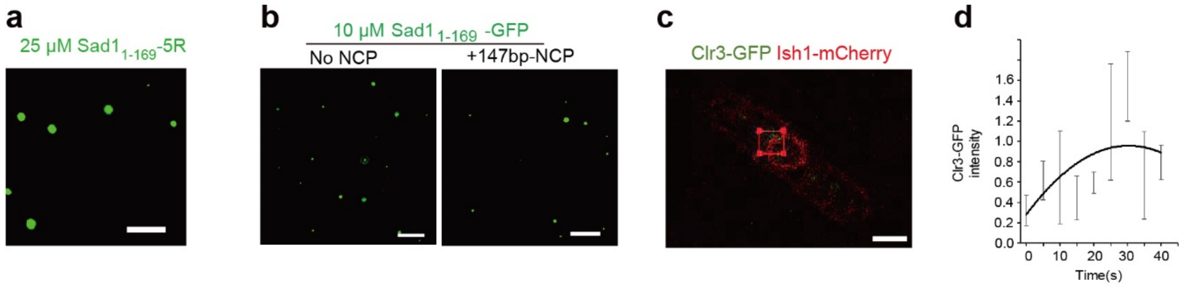

Supplementary Figure 12. Phase separation of $\mathbf{Sad1_{1\text{-}169}}$ and Clr3.

a. Fluorescence image of droplet formation of Sad1-5R-GFP (25 μM) in droplet formation buffer. Sad1-5R can still form droplets similar to wild-type Sad1. Scale bar, 10 μm.

b. Fluorescence images of a mixture of $Sad1_{1\text{-}169}$-GFP and 147bp nucleosome. The nucleosome had no effect on droplet size of Sad1. Scale bar, 10 μm.

c. Fluorescence image of Clr3-GFP region used for FRAP experiment. Clr3-GFP expressed from its native promoter forms distinct puncta. The bleached region is indicated with a red square. The nuclear envelope was represented by Ish1-mCherry. Scale bar, 2 μm.

d. The plot of normalized recovery of the Clr3-GFP signal after photobleaching. Data are presented as mean ± SD from n=3 independent experiments. Source data are provided as a Source Data file.

Supplementary Table 1. Data collection and refinement statistics for the crystal structure.

	$Sad1_{110\text{-}126}$-H2AB [a]
**Data collection**	
Space group	$P2_1$
Cell dimensions	
a, b, c (Å)	42.80, 61.98, 76.02
α, β, γ (°)	90, 101.89, 90
Wavelength (Å)	0.9778
Resolution (Å)	50.00-2.15
R_{merge}	0.123 (0.552)[b]
I /σI	12.3 (3.1)
Completeness (%)	99.8 (99.7)
Redundancy	5.5 (5.5)
**Refinement**	
Resolution (Å)	37.19-2.15
No. reflections	21036
R_{work}/R_{free} (%)	19.8/24.8
No. atoms	
Protein	2854
Water	97
B-factors (Å^2)	
Protein	29.4
Water	35.8
R.m.s deviations	
Bond lengths (Å)	1.101
Bond angles (°)	0.009
Ramachandran statistics	
Favored (%)	97.21
Allowed (%)	2.79
Outliers (%)	0.0

[a] The data was collected from one crystal.

[b] Values in parentheses are for the highest-resolution shell.

Supplementary Table 2. Strains used in this study

Strain	Genotype
FL598	h^{+} otr3L-r2::ura4^{+} leu1-32 ade6-M210 his3-D1 ura4-D18
FL602	h^{-} otr3L-r10::ura4^{+} leu1-32 ade6-M210 his3-D1 ura4-D18
FL734	h^{+} Ch16-m23::LEU2 tel::ade6^{+} leu1-32 ade6ΔN/N ura4-DS/E
FL920	h^{-} sad1::sad1-GFP-kanMX6 leu1-32 ade6-M210 ura4-D18
FL922	h^{-} sad1::sad1-5R-GFP-kanMX6 leu1-32 ade6-M210 ura4-D18
FL923	h^{-} sad1::sad1-D21-GFP-kanMX6 leu1-32 ade6-M210 ura4-D18
FL924	h$^{?}$ sad1::sad1-GFP-kanMX6 ish1^{+}-mCherry-hph leu1-32 ura4-D18
FL925	h$^{?}$ sad1::sad1-5R-GFP-kanMX6 ish1^{+}-mCherry-hph leu1-32 ura4-D18
FL926	h^{-} sad1-5R leu1-32 ade6-M210 ura4-D18
FL927	h^{-} sad1-D21 leu1-32 ade6-M210 ura4-D18
FL928	h$^{?}$ sad1-5R taz1-GFP::kanR ish1^{+}-mCherry-hph leu1-32 ura4-D18
FL929	h$^{?}$ sad1-D21 taz1-GFP::kanR ish1^{+}-mCherry-hph leu1-32 ura4-D18
FL930	h$^{?}$ his2[::kanr-ura4^{+}-lacOp] his7^{+}::lacI-GFP ish1^{+}-mCherry-hph leu1-32 ura4-D18
FL931	h$^{?}$ sad1-5R his2[::kanr-ura4^{+}-lacOp] his7^{+}::lacI-GFP ish1^{+}-mCherry-hph leu1-32 ura4-D18
FL933	h$^{?}$ sad1::sad1-GFP-kanMX6 tel2L::ura4^{+} ura4-D18/ ura4-DS/E?
FL934	h$^{?}$ sad1::sad1-5R-GFP-kanMX6 tel2L::ura4^{+} ura4-D18/ ura4-DS/E?
FL935	h$^{?}$ sad1::sad1-GFP-kanMX6 mat3-M::ura4^{+} leu1-32 ura4-D18/ ura4-DS/E?
FL936	h$^{?}$ sad1::sad1-5R-GFP-kanMX6 mat3-M::ura4^{+} leu1-32 ura4-D18/ ura4-DS/E?
FL937	h$^{?}$ sad1-5R ade6^{+}::cnp1-GFP leu1-32 ade6-M210 ura4D-18
FL938	h$^{?}$ sad1-D21 ade6^{+}::cnp1-GFP leu1-32 ade6-M210 ura4D-18
FL939	h$^{?}$ sad1::sad1-5R-GFP-kanMX6 otr3L-r2::ura4^{+} leu1-32 ade6-M210 ura4-D18
FL940	h$^{?}$ sad1::sad1-5R-GFP-kanMX6 otr3L-r10::ura4^{+} leu1-32 ade6-M210 ura4-D18
FL942	h$^{?}$ Ch16-m23::LEU2 tel::ade6^{+}sad1::sad1-5R-GFP-kanMX6 leu1-32 ade6ΔN/N
FL944	h$^{?}$ sad1::sad1-5R-HA-KanMX6 htb1-FLAG-nat ura4-D18
FL945	h$^{?}$ htb1-FLAG-nat ura4-D18
FL604	h$^{?}$ dos1Δ::KanR otr3LR2::ura4^{+} ura4D18 leu1-32 ade6-210 his3D1
JG1	h$^{?}$ sad1-5R-GFP::KanR otr3LR2::ura4^{+} ura4D18 leu1-32 ade6-210 his3D1
FL961	h$^{?}$ sad1-F118R-GFP::KanR^{+} otr3LR2::ura4 ura4D18 leu1-32 ade6-210 his3D1

JG2	$h^{?}$ *dos1Δ::KanR otr3LR10::ura4*$^{+}$ *ura4D18 leu1-32 ade6-210 his3D1*
JG3	$h^{?}$ *sad1-5R-GFP::KanR otr3LR10::ura4*$^{+}$ *ura4D18 leu1-32 ade6-210 his3D1*
FL962	$h^{?}$ *sad1-F118R-GFP::KanR otr3LR10::ura4*$^{+}$ *ura4D18 leu1-32 ade6-210 his3D1*
JG5	$h^{?}$ *dos1Δ::KanR mat3-M::ura4*$^{+}$ *ade6-210 leu1-32 ura4-DS/E*
JG6	$h^{?}$ *sad1-5R-GFP::KanR mat3-M::ura4*$^{+}$ *ade6-210 leu1-32 ura4-DS/E*
JG7	$h^{?}$ *sad1-F118R::KanR mat3-M::ura4*$^{+}$ *ade6-210 leu1-32 ura4-DS/E*
JG8	$h^{?}$ *dos1Δ::KanR TEL2L-ura4*$^{+}$ *ade6-210 his3D1 leu1-32 ura4DS/E*
JG9	$h^{?}$ *sad1-5R::KanR TEL2L-ura4*$^{+}$ *ade6-210 his3D1 leu1-32 ura4DS/E*
FL964	$h^{?}$ *sad1-F118R::KanR TEL2L-ura4*$^{+}$ *ade6-210 his3D1 leu1-32 ura4DS/E*
JG10	$h^{?}$ *sad1-D21-GFP::KanR TEL2L-ura4*$^{+}$ *ade6-210 his3D1 leu1-32 ura4DS/E*
JG12	$h^{?}$ *dos1Δ::KanR leu1-32 ura4-D18 ade6ΔN/N ch16-M23::Leu2*$^{+}$ *tel::ade6*$^{+}$
FL965	$h^{?}$ *sad1-F118R-GFP::KanR leu1-32 ura4-D18 ade6ΔN/N ch16-M23::Leu2*$^{+}$ *tel::ade6*$^{+}$
FL1049	h^{90} *clr3Δ-KanR mat3-M::ura4*$^{+}$ *ade6-210 leu1-32 ura4-DS/E*
FL1050	h^{90} *sir2Δ-KanR mat3-M::ura4*$^{+}$ *ade6-210 leu1-32 ura4-DS/E*
FL1051	$h^{?}$ *clr3Δ-KanR sad1-5R-NatR mat3-M::ura4*$^{+}$ *ade6-210 leu1-32 ura4-DS/E*
FL1052	$h^{?}$ *sir2Δ-KanR sad1-5R-NatR mat3-M::ura4*$^{+}$ *ade6-210 leu1-32 ura4-DS/E*
FL1053	$h^{?}$ *sad1-5R-NatR mat3-M::ura4*$^{+}$ *ade6-210 leu1-32 ura4-DS/E*
FL1054	h^{90} *clr3Δ-KanR TEL2L-ura4*$^{+}$ *ade6-210 leu1-32 ura4-DS/E*
FL1055	h^{90} *sir2Δ-KanR TEL2L-ura4*$^{+}$ *ade6-210 leu1-32 ura4-DS/E*
FL1056	$h^{?}$ *clr3Δ-KanR sad1-5R-NatR TEL2L-ura4*$^{+}$ *ade6-210 leu1-32 ura4-DS/E*
FL1057	$h^{?}$ *sir2Δ-KanR sad1-5R-NatR TEL2L-ura4*$^{+}$ *ade6-210 leu1-32 ura4-DS/E*
FL1058	$h^{?}$ *sad1-5R-NatR TEL2L-ura4*$^{+}$ *ade6-210 leu1-32 ura4-DS/E*
FL1059	$h^{?}$ *sad1::sad1-HA-KanMX6 htb1-FLAG-nat ura4-D18*
