# Supplementary material for: The SUN-family protein Sad1 mediates heterochromatin spatial organization through interaction with histone H2A-H2B
Source: Nat Commun. 2024 May 21;15:4322. doi: 10.1038/s41467-024-48418-7 (PMC11109203; doi:10.1038/s41467-024-48418-7)
Supplement: Supplementary file 3 — Description of Additional Supplementary Files [file 41467_2024_48418_MOESM3_ESM.pdf]

Supplementary Movie 1.

Description:

Time-lapse fluorescence microscopy of Sad1-GFP under its native promoter shows the dynamic movement of Sad1-GFP puncta on the nuclear envelope. The duration of the video is 40 min, and the recording time interval is 2 min/frame. The display rate is 3 frames/s. Scale bar, 1 μm.
