# Supplementary material for: The SUN-family protein Sad1 mediates heterochromatin spatial organization through interaction with histone H2A-H2B
Source: Nat Commun. 2024 May 21;15:4322. doi: 10.1038/s41467-024-48418-7 (PMC11109203; doi:10.1038/s41467-024-48418-7)
Supplement: Supplementary file 5 — Reporting Summary [file 41467_2024_48418_MOESM5_ESM.pdf]

nature portfolio

Corresponding author(s): Yong Chen

Last updated by author(s): 2024/03/18

Reporting Summary

Nature Portfolio wishes to improve the reproducibility of the work that we publish. This form provides structure for consistency and transparency in reporting. For further information on Nature Portfolio policies, see our Editorial Policies and the Editorial Policy Checklist.

Statistics

For all statistical analyses, confirm that the following items are present in the figure legend, table legend, main text, or Methods section.

n/a	Confirmed	
☐	☒	The exact sample size (*n*) for each experimental group/condition, given as a discrete number and unit of measurement
☐	☒	A statement on whether measurements were taken from distinct samples or whether the same sample was measured repeatedly
☐	☒	The statistical test(s) used AND whether they are one- or two-sided<br>*Only common tests should be described solely by name; describe more complex techniques in the Methods section.*
☒	☐	A description of all covariates tested
☒	☐	A description of any assumptions or corrections, such as tests of normality and adjustment for multiple comparisons
☐	☒	A full description of the statistical parameters including central tendency (e.g. means) or other basic estimates (e.g. regression coefficient) AND variation (e.g. standard deviation) or associated estimates of uncertainty (e.g. confidence intervals)
☐	☒	For null hypothesis testing, the test statistic (e.g. *F*, *t*, *r*) with confidence intervals, effect sizes, degrees of freedom and *P* value noted<br>*Give P values as exact values whenever suitable.*
☒	☐	For Bayesian analysis, information on the choice of priors and Markov chain Monte Carlo settings
☒	☐	For hierarchical and complex designs, identification of the appropriate level for tests and full reporting of outcomes
☒	☐	Estimates of effect sizes (e.g. Cohen's *d*, Pearson's *r*), indicating how they were calculated

Our web collection on statistics for biologists contains articles on many of the points above.

Software and code

Policy information about availability of computer code

Data collection	ITC assays were performed using a MicroCal ITC200 system (GE Healthcare). All MST measurements were performed on a Monolith NT.115 system (Nano Temper Technologies, Germany). NanoDSF measurements were carried out on a Prometheus NT.48 system (NanoTemper Technologies, Germany).The crystal data were collected at the beamline BL19U1/BL18U1 of Shanghai Synchrotron Radiation Facility. Imaging of the in vitro LLPS droplets was captured by a Zeiss LSM 710 microscope (Zeiss, Germany). Yeast cells were imaged using a BX53 fluorescence microscope (Olympus, Japan) or the Delta Vision System (Applied Precision, USA). The cellular FRAP experiments were performed using the Zeiss Airyscan Confocal system (Zeiss, Germany).
Data analysis	PyMOL 1.5.0, GraphPad Prism 8.0, Phenix 1.18.2, Coot 0.8.2, HKL2000_716, Origin 9.0, SoftWoRX 2.50,ImageJ v1.53a

For manuscripts utilizing custom algorithms or software that are central to the research but not yet described in published literature, software must be made available to editors and reviewers. We strongly encourage code deposition in a community repository (e.g. GitHub). See the Nature Portfolio guidelines for submitting code & software for further information.

Data

Policy information about availability of data

All manuscripts must include a data availability statement. This statement should provide the following information, where applicable:

- Accession codes, unique identifiers, or web links for publicly available datasets
- A description of any restrictions on data availability
- For clinical datasets or third party data, please ensure that the statement adheres to our policy

Coordinates and structure factors of Sad1HBM-H2AB has been deposited in the Protein Data Bank under accession codes 7YBF [http://doi.org/10.2210/pdb7YBF/pdb]. The following structures were used in the paper for structural analyses: 6AE8 [http://doi.org/10.2210/pdb6AE8/pdb], 4WNN [http://doi.org/10.2210/pdb4WNN/pdb], 4CAY [http://doi.org/10.2210/pdb4CAY/pdb], 4M6B [http://doi.org/10.2210/pdb4M6B/pdb]. Source data are provided with this paper. All the protein constructs are available from Dr. Yong and all the yeast strains are available from Dr. Fei Li upon request.

Research involving human participants, their data, or biological material

Policy information about studies with human participants or human data. See also policy information about sex, gender (identity/presentation), and sexual orientation and race, ethnicity and racism.

Reporting on sex and gender	NA
Reporting on race, ethnicity, or other socially relevant groupings	NA
Population characteristics	NA
Recruitment	NA
Ethics oversight	NA

Note that full information on the approval of the study protocol must also be provided in the manuscript.

Field-specific reporting

Please select the one below that is the best fit for your research. If you are not sure, read the appropriate sections before making your selection.

☒ Life sciences ☐ Behavioural & social sciences ☐ Ecological, evolutionary & environmental sciences

For a reference copy of the document with all sections, see nature.com/documents/nr-reporting-summary-flat.pdf

Life sciences study design

All studies must disclose on these points even when the disclosure is negative.

Sample size	We used the sample size based on the common setups in similar researches to reach the balance of practical feasibility and scientific rigor. The exact sample size used is been described in figure legend of each figure.
Data exclusions	NA.
Replication	The biochemical assays, including pull-down assay, ITC, and Co-IP assays were performed at least twice and similar results were obtained, so the representative results were shown. For imaging experiments, replication numbers were indicated in the figure legends or the method section under“ Statistics and reproducibility” section.
Randomization	For imaging experiments, cells and regions for imaging were selected randomly. For other biochemical assays, randomization was not performed.
Blinding	During the experiments, researchers were not blinded to the identity of the samples, but findings were replicated and confirmed by multiple experiments or different researchers.

Reporting for specific materials, systems and methods

We require information from authors about some types of materials, experimental systems and methods used in many studies. Here, indicate whether each material, system or method listed is relevant to your study. If you are not sure if a list item applies to your research, read the appropriate section before selecting a response.

Materials & experimental systems

n/a	Involved in the study
☐	☒ Antibodies
☐	☒ Eukaryotic cell lines
☒	☐ Palaeontology and archaeology
☒	☐ Animals and other organisms
☒	☐ Clinical data
☒	☐ Dual use research of concern
☒	☐ Plants

Methods

n/a	Involved in the study
☒	☐ ChIP-seq
☒	☐ Flow cytometry
☒	☐ MRI-based neuroimaging

Antibodies

Antibodies used

anti-FLAG primary antibody (F1804, Sigma, USA), anti-GFP HRP (ab190584, Abcam, UK), anti-GFP primary antibody (ab290, Abcam, UK) , anti-HA primary antibody (12CA5, Roche, USA), anti-TAP primary antibody (P1291, Sigma, USA), and Goat Anti-Mouse IgG H&L (HRP) (ab6789, Abcam,UK)

Validation

These antibodies are commercial available and have been validated by manufactures.
Anti-Flag (F1804):The ANTI-FLAG M2 mouse, affinity purified monoclonal antibody binds to fusion proteins containing a FLAG peptide sequence. The antibody recognizes the FLAG peptide sequence at the N-terminus, Met-N-terminus,C-terminus, and internal sites of the fusion protein. Host: mouse. For highly sensitive and specific detection of FLAG fusion proteins by immunoblotting, immunoprecipitation (IP) immunohistochemisty, immunofluorescence, and immunocyotchemistry. Optimized for single banded detection of FLAG fusion proteins in mammalian, plant, and bacterial expression systems.The detailed information can be found from https://www.sigmaaldrich.com/US/en/product/sigma/f1804.
Anti-GFP (ab290, Abcam): Anti-GFP antibody (ab290) is a rabbit polyclonal antibody that gives a stronger signal than other anti-GFP antibodies available. Species independent. GFP antibody is reactive against all variants of Aequorea victoria GFP such as S65T-GFP, RS-GFP, YFP, CFP, RFP and EGFP. Suitable for: ELISA, IHC-Fr, ICC, IHC-P, IP, WB, IHC-FoFr, IHC-FrFl, Electron Microscopy. The detailed information can be found from https://www.abcam.com/products/primary-antibodies/gfp-antibody-ab290.html.

Anti-GFP HRP (ab190584, Abcam, UK): is a HRP Rabbit monoclonal [E385] to GFP produced recombinantly (animal-free) for high batch-to-batch consistency and long term security of supply. Suitable for WB. Species independent. The detailed information can be found from: https://www.abcam.com/products/primary-antibodies/hrp-gfp-antibody-e385-ab190584.html

Anti-HA (12CA5, Roche): Anti-HA High Affinity is a monoclonal antibody to the HA-peptide (clone 3F10). Anti-HA High Affinity recognizes the HA peptide sequence (YPYDVPDYA). Suitable for Dot blots, ELISA, Immunocytochemistry,Immunoprecipitation, and western blot.
The detailed information can be found from: https://www.sigmaaldrich.com/US/en/product/roche/roahaha

Anti-TAP antibody (P1291, Sigma): Peroxidase Anti-Peroxidase (PAP) soluble complex is prepared by a modification of the method of Sternberger using specific polyclonal or monoclonal antibodies to horseradish peroxidase and highly purified enzyme. Since the enzyme is not covalently coupled to the antibody, the soluble complex retains its enzyme and antibody reactivity with distinct advantages over enzyme-conjugated antibodies. Peroxidase Anti-Peroxidase Soluble Complex antibody produced in rabbit has been used in immunoblotting, immunoprecipitation, and immunohistochemical staining. The detailed information can be found from: https://www.sigmaaldrich.com/US/en/product/sigma/p1291.

Goat Anti-Mouse IgG H&L (HRP) (ab6789, abcam): an HRP-conjugated polyclonal secondary antibody. Host species: Goat. Target species: Mouse. Target: IgG heavy and light chains. Validated in IP, Dot, IM, IHC-Fr, WB, IHC-P, ELISA, ICC. Ideal for western blot. The detailed information can be found from:
https://www.abcam.com/en-by/products/secondary-antibodies/goat-mouse-igg-h-l-hrp-ab6789

Eukaryotic cell lines

Policy information about cell lines and Sex and Gender in Research

Cell line source(s)

S.pombe yeast cell lines are constructed by Fei Li lab. The full list of strains used in this study has been included as Supplementary Table 2.

Authentication

Describe the authentication procedures for each cell line used OR declare that none of the cell lines used were authenticated.

Mycoplasma contamination

Confirm that all cell lines tested negative for mycoplasma contamination OR describe the results of the testing for mycoplasma contamination OR declare that the cell lines were not tested for mycoplasma contamination.

Commonly misidentified lines
(See ICLAC register)

Name any commonly misidentified cell lines used in the study and provide a rationale for their use.

Plants

Seed stocks	*Report on the source of all seed stocks or other plant material used. If applicable, state the seed stock centre and catalogue number. If plant specimens were collected from the field, describe the collection location, date and sampling procedures.*
Novel plant genotypes	*Describe the methods by which all novel plant genotypes were produced. This includes those generated by transgenic approaches, gene editing, chemical/radiation-based mutagenesis and hybridization. For transgenic lines, describe the transformation method, the number of independent lines analyzed and the generation upon which experiments were performed. For gene-edited lines, describe the editor used, the endogenous sequence targeted for editing, the targeting guide RNA sequence (if applicable) and how the editor was applied.*
Authentication	*Describe any authentication procedures for each seed stock used or novel genotype generated. Describe any experiments used to assess the effect of a mutation and, where applicable, how potential secondary effects (e.g. second site T-DNA insertions, mosiacism, off-target gene editing) were examined.*
